# Supplementary material for: Femtosecond time-resolved two-photon photoemission studies of ultrafast carrier relaxation in Cu2O photoelectrodes
Source: Nat Commun. 2019 May 8;10:2106. doi: 10.1038/s41467-019-10143-x (PMC6506537; doi:10.1038/s41467-019-10143-x)
Supplement: Supplementary file 1 — Supplementary Information [file 41467_2019_10143_MOESM1_ESM.pdf]

# Femtosecond time-resolved two-photon photoemission studies of ultrafast carrier relaxation in $Cu_2O$ photoelectrodes

Borgwardt et al.

## Supplementary Figures

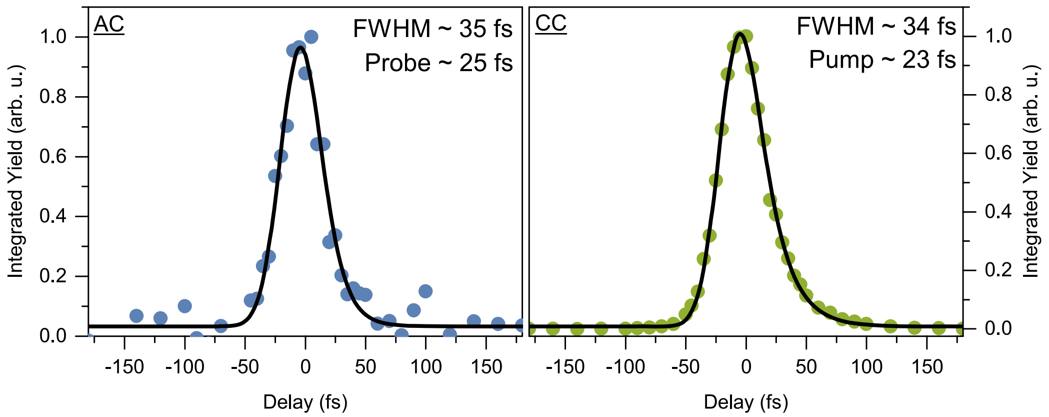


**Supplementary Figure 1. Measurement and optimization of the instrument response function (IRF).** Autocorrelation (AC, left) and crosscorrelation (CC, right) traces obtained at single crystal Cu (111) samples. To achieve satisfactory time resolution, the pump and probe pulses were compressed with prism pairs. To verify optimized temporal resolution of the 2PPE set-up, auto- (AC) and cross correlation (CC) measurements were performed on Cu (111) single crystals using the response of a two-photon process from the occupied surface-state mediated via a virtual intermediate state in the sp-band gap[1]. Pulse durations of 23 fs for the pump and 25 fs for the probe pulses were found.

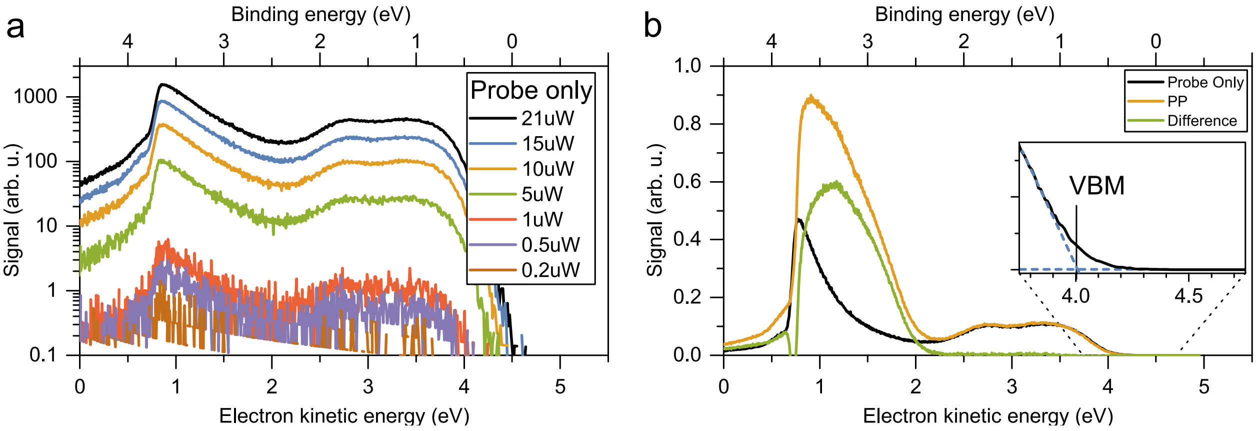


**Supplementary Figure 2. Single-color steady state spectra.** (a) Single-color steady state spectra of the reconstructed sample for different probe intensities plotted on a logarithmic scale. (b) Two-color spectrum ($\Delta t=0$) (PP, yellow) and single-color probe spectrum (black) of the reconstructed sample. The difference spectrum (green line) depicts the signal originating from the two photon transitions involving one pump and one probe photon. The inset shows the measurement of the valence-band maximum (VBM).

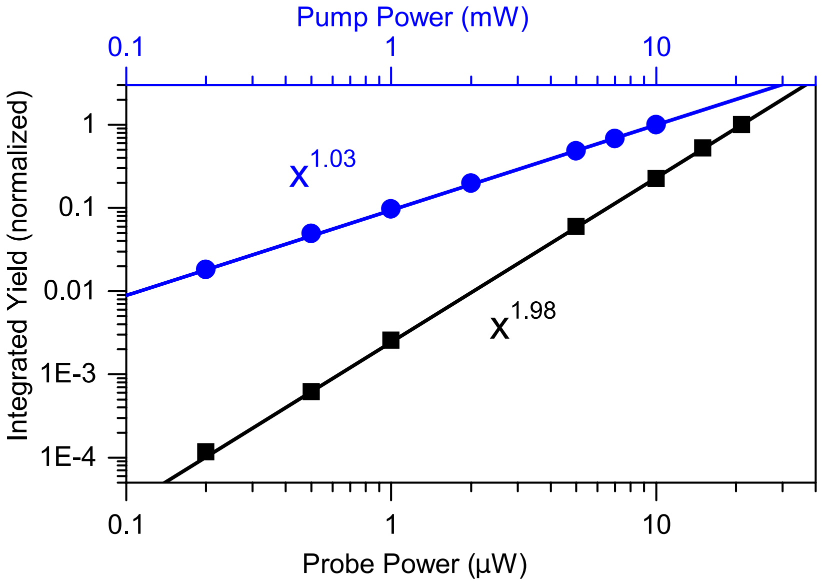


**Supplementary Figure 3. Power dependence**. Total electron yield (TEY) as a function of laser power for the probe only (black, bottom scale) and for pump-probe spectra (blue, top scale). In the latter case the TEY was corrected by the yield measured when only the probe beam was used.

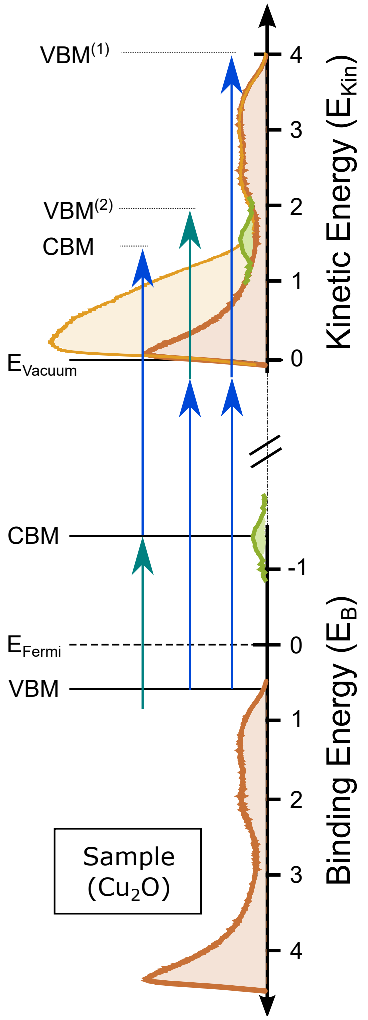


**Supplementary Figure 4. Excitation scheme for two-photon photoemission.** The schematic shows the photoemission process involving multiple photons. In a single-color experiment (probe only, two blue arrows, right) a two-photon transition lifts an electron from the VBM 0.5 eV below the Fermi level to above the vacuum level reaching the VBM$^{(1)}$ as indicated level on the kinetic energy scale. For the two-color experiment (probe and pump, blue and green arrow, center) the VBM is projected onto a lower level indicated as VBM$^{(2)}$ due to the lower combined photon energy. Both cases are mediated via virtual states and the energetic band alignment of the occupied states (valence band) is measured. In contrast, two photon transitions via resonant intermediates probe the energetic structure of the unoccupied states. This process is illustrated by

the two-photon transition (pump and probe, green and blue arrow, left) lifting first an electron from the VB above the band gap and subsequently exciting the photoelectron above the vacuum level.

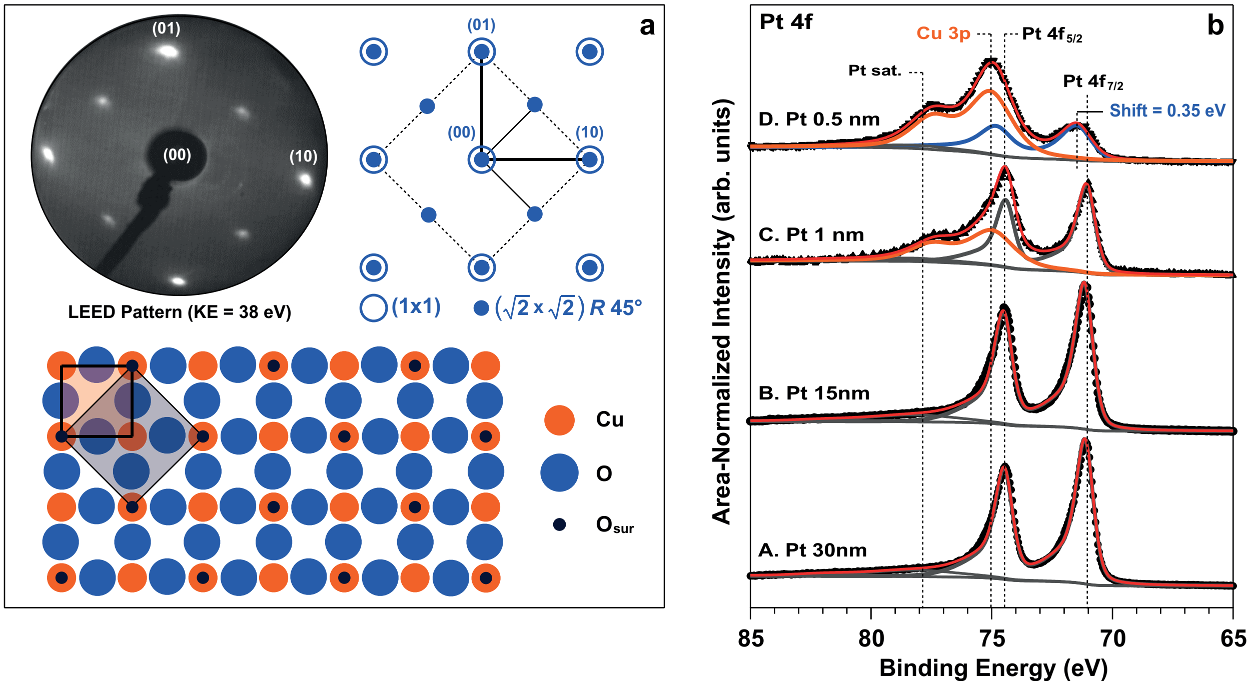


**Supplementary Figure 5. Low-energy electron diffraction (LEED) and X-ray photoelectron spectroscopy.** (a) Structure of the reconstructed $Cu_2O$ (100) surface. LEED data showed that the reconstructed surface exhibited a (1×1) periodicity with ½ monolayer of terminating atomic oxygen adsorbed as a c(2×2) structure (or, in Wood notation, as √2×√2 R45°); (b) Pt 4f photoemission spectra, acquired for different Pt thicknesses on the $Cu_2O$ (100) surface (hν (Al Kα) = 1486.7 eV).

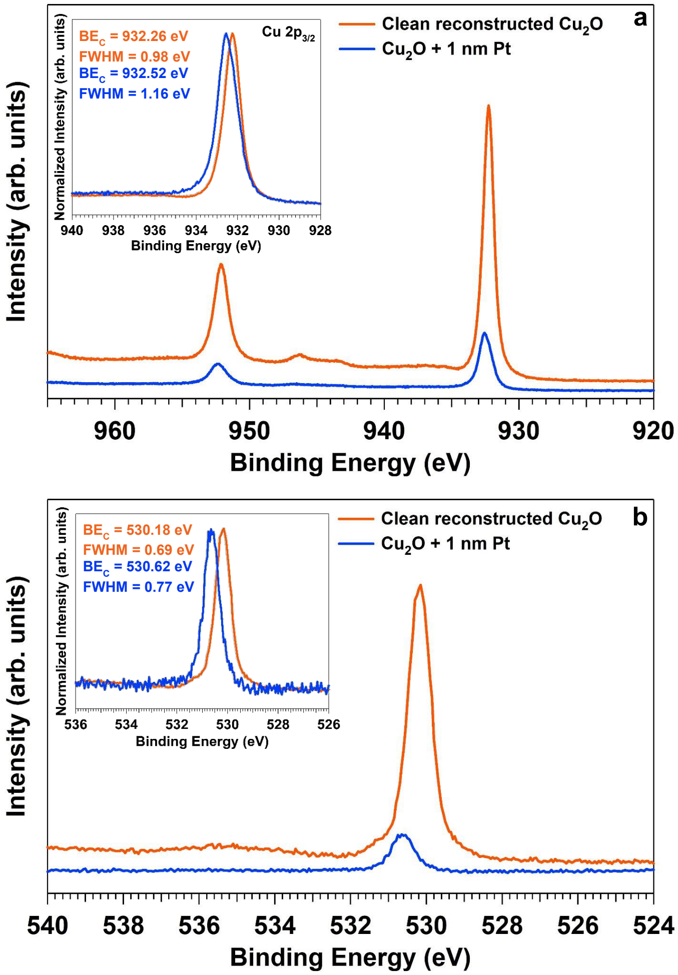


**Supplementary Figure 6. Core-level analysis of the $Cu_2O$ (100) surface for pristine conditions (clean and reconstructed surface) and after the deposition of 1.0 nm of Pt.** The analysis was performed using the Al Kα photon energy (hv = 1486.7 eV). (a) Cu 2p, (b) O 1s.

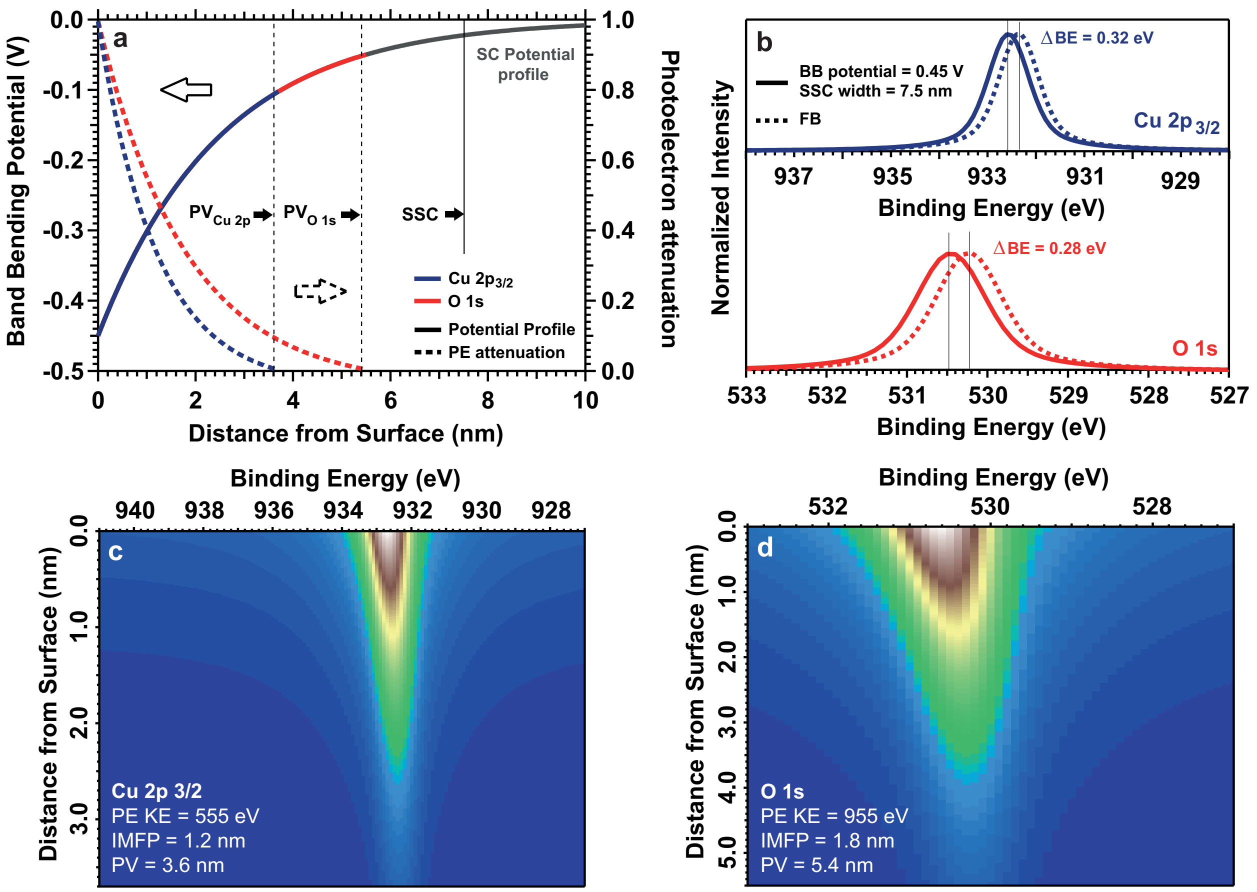


**Supplementary Figure 7. Simulation of the Cu 2p3/2 and O 1s core levels within the semiconductor.** The simulations have been performed using a full-width at half-maximum of 1 eV for the single spectral components, described by a Voigt line shape with a Gaussian-to-Lorentzian ratio (G/L) equal to 0.75. (a) Semiconductor (SC) potential profile and potential profiles probed using the Cu $2p_{3/2}$ and O 1s core levels, with a band bending (BB) potential of 0.45 V and a space charge layer of 7.5 nm. The SC potential profile has been modelled with an exponential function, to take into account temperature effects; (b) simulated Cu $2p_{3/2}$ and O 1s core levels under BB and flat-band conditions. When a downward BB is present, the core level binding energies shift to higher values (ΔBE Cu 2p = 0.32 eV, ΔBE O 1s = 0.28 eV); (c), (d) 2D plots reporting the simulated Cu $2p_{3/2}$ and O 1s core levels, respectively, as a function of the distance from the surface (i.e. as a function of the probed SC potential profile and photoelectron

exponential decay). SC: semiconductor; SSC: semiconductor space charge; FB: flat band conditions; BB: band bending conditions; PE KE: photoelectron kinetic energy; IMFP: inelastic mean free path; PV: probed volume.

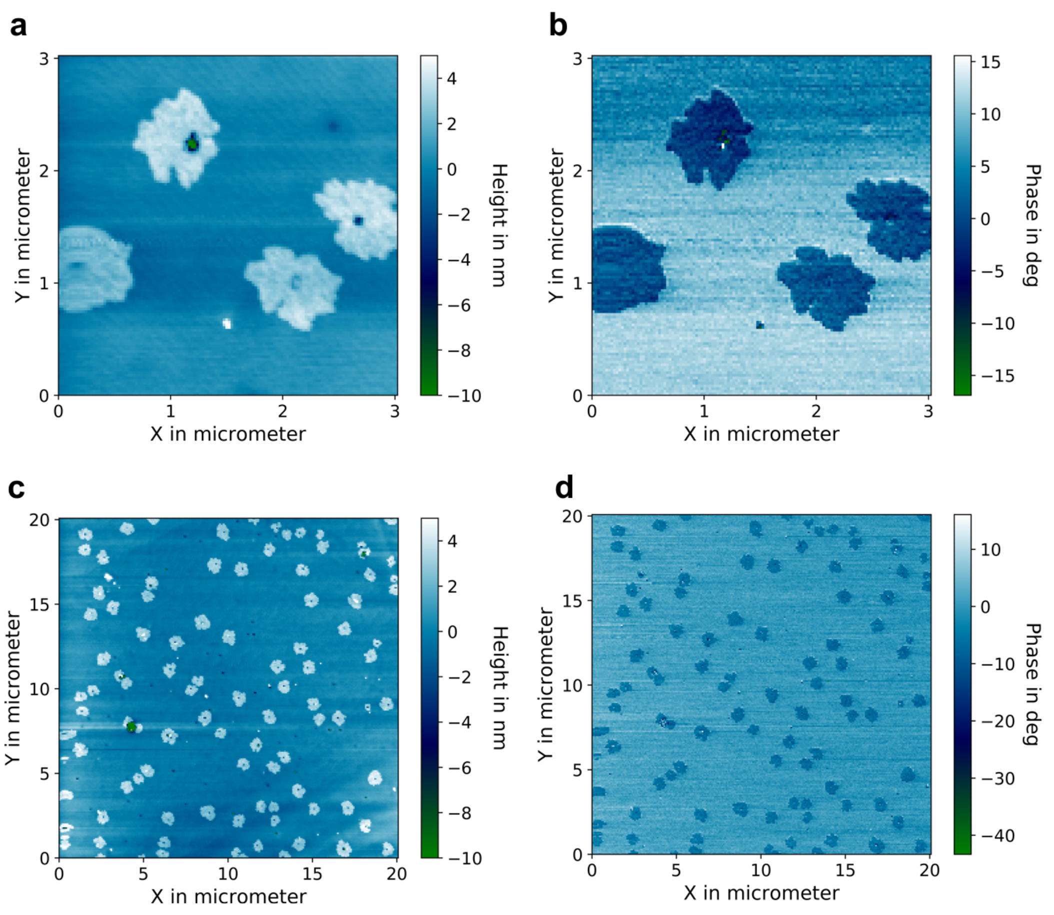


**Supplementary Figure 8. Atomic force microscopy (AFM).** AFM height (a,c) and phase (b,d) information for the Pt covered reconstructed $Cu_2O$ (100) surface.

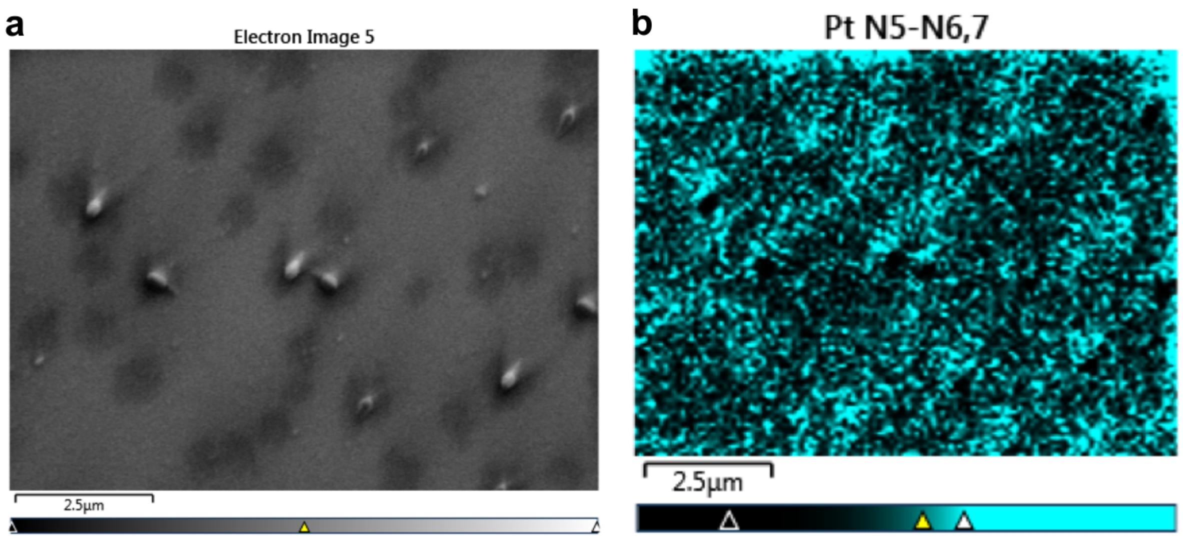


**Supplementary Figure 9. Scanning electron microscopy and elemental distribution mapping (EDX).** SEM image (a) and EDX elemental distribution map using the Pt N line (b), both acquired on the identical area of the Pt covered reconstructed $Cu_2O$ (100) surface.

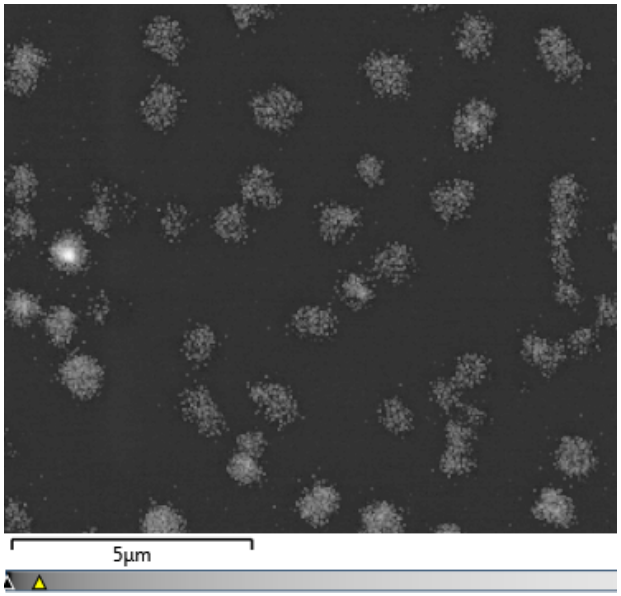


**Supplementary Figure 10. SEM image of the Pt covered reconstructed $Cu_2O$ (100) surface.**

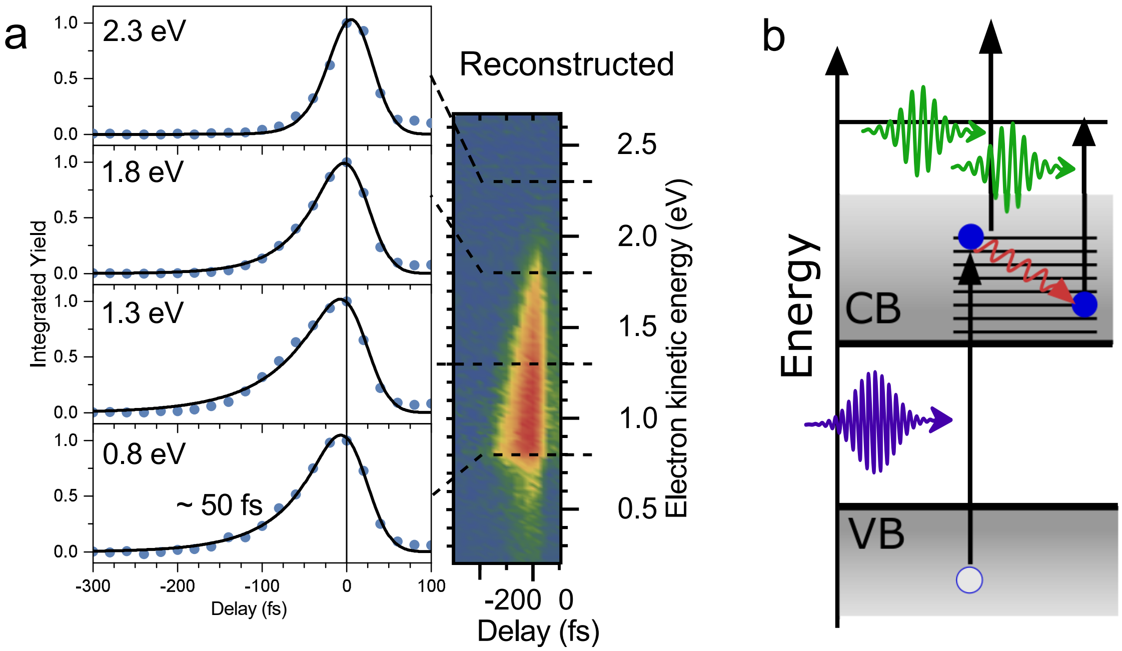


**Supplementary Figure 11. Hot carrier cooling at negative time delays.** (a) Color map of the transient photoemission signal as a function of the electron kinetic energy and the pump probe time delay in the vicinity of zero time delay. Spectral cuts at the specified electron kinetic energies are also shown, as indicated in each panel. (b) Schematic illustration of the hot-carrier cooling process measured by the 2PPE technique. In the vicinity of zero time delay (±100 fs), the transient signal (TS) exhibited a strong contribution at kinetic energies between 0.5 and 2.5 eV (Supplementary Figure 5a). The symmetric part of this feature can be attributed to the cross-correlation (CC) signal represented by a sharp falling edge at positive time delays (pump first). In contrast, the signal exhibited increased asymmetry for lower kinetic energies and negative time delays (probe incident first). This asymmetric portion of the signal can consistently be ascribed to thermal cooling of an initially hot electron distribution that subsequently relaxes towards the conduction-band minimum. Due to the much higher probe photon energy compared to the electronic band gap of $Cu_2O$ (2.1 eV), photoexcitation is expected to produce a

pronounced hot carrier distribution well above the band edges. Typically, carrier cooling processes occur on timescales from tens to hundreds of fs, which is on the order of the system response of the 2PPE measurement[2,3]. The cooling process of the electron distribution involves a continuum of states, so the effective lifetime of lower lying states (higher binding energy) increases due to the subsequent population from states above. This process is expected to produce an increasing asymmetry of the transient signal for decreasing electron kinetic energies in accord with observations (Supplementary Figure 5a). An effective lifetime of about (50±10) fs was measured for the lowest kinetic energy of 0.8 eV probed experimentally.

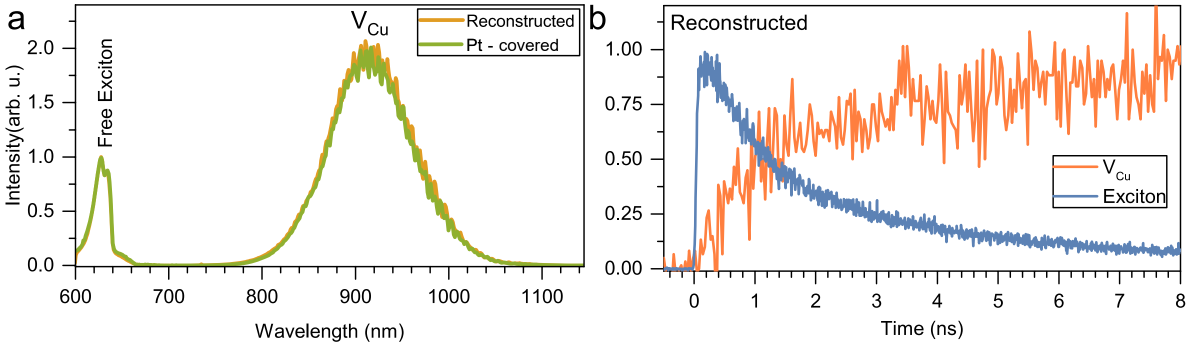


**Supplementary Figure 12. Photoluminescence (PL) and time-resolved Photoluminescence (trPL).** (a) Photoluminescence spectra at different stages of $Cu_2O$ (100) sample preparation (reconstructed, Pt-covered). The pump beam (494 nm) was used to excite the sample. (b) Time-resolved PL at the reconstructed $Cu_2O$ (100) sample for the two spectral regions encompassing the exciton PL (short pass filter at 650 nm) as well as the defect emission (long pass at 900 nm). The experiments were conducted at different stages of sample preparation (reconstructed, Pt-covered) while maintaining UHV conditions throughout.

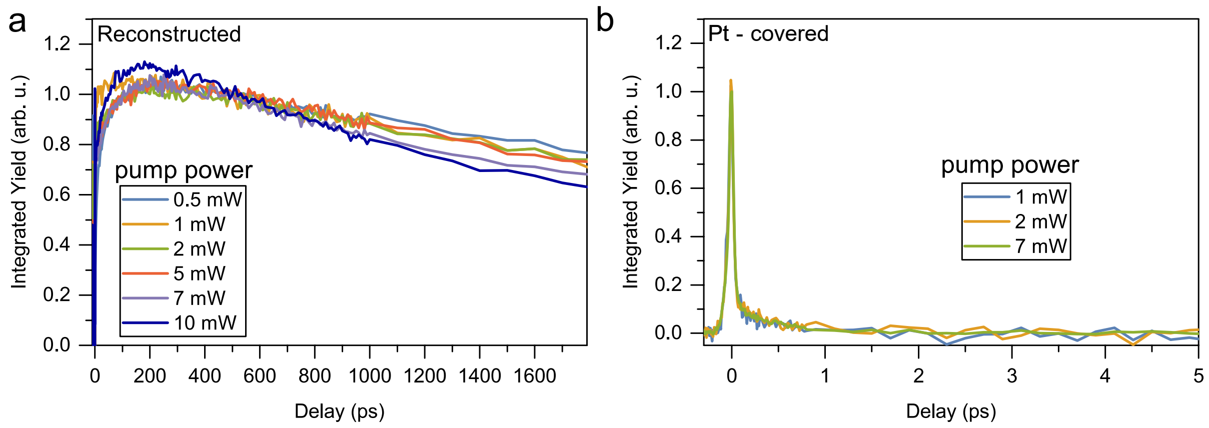


**Supplementary Figure 13. Power dependence of the total electron yield (TEY).** Intensity-dependent 2PPE signal at the reconstructed $Cu_2O$ (a) and Pt-deposited $Cu_2O$ sample (b). Intensity-dependent measurements were performed on the reconstructed $Cu_2O$ and Pt-deposited $Cu_2O$ sample. In the case of the reconstructed sample, biexponential fits revealed that the time constant of the fast rise component (~1 ps) decreased with increasing pump pulse intensity, whereas the second rise component (~80 ps) was independent of the pump pulse intensity. No pump power dependence was observed for the Pt-deposited $Cu_2O$ sample.

## Supplementary Notes

### Supplementary Note 1

Single-color (steady state spectra) spectra at the reconstructed sample: Intensity dependent measurements were performed to understand the energetic band alignment (occupied states) and to analyze the single-color background that contributed to the 2PPE signal. When the pump beam only with a photon energy of 2.5 eV was used, a negligible amount of photoelectrons were detected because at least three of these photons are required to cross the ionization threshold of >5.1 eV[4]. In contrast, only two probe-photons of 4.5 eV are required to cross the ionization threshold, resulting in much larger signal intensities (see Supplementary Figure 4, two blue arrows). The probe beam itself may therefore contribute a delay-independent spectral portion to the transient signal of the multi-color pump-probe experiments. To determine this contribution, probe spectra were obtained for different intensities (Supplementary Figure 2a), and the corresponding total electron yield (TEY) was plotted (Supplementary Figure 3 - black line). A quadratic dependence of the TEY was found, confirming that the photoionization process involved a two-photon transition (hν = 9.0 eV). The Fermi edge ($E_F = E_B = 0$ eV) of a metallic copper reference sample allowed further calibration of the binding energy $E_B$ scale, as follows:

$$\boldsymbol{E_{Kin} = h\nu - E_B - \phi} \tag{1}$$

where $E_{Kin}$ is the directly measured electron kinetic energy and Φ the work function of the spectrometer. As an example, the binding energy scale that is valid for this specific transition (two probe photons, hν = 9.0 eV) is indicated by the top scales of Supplementary Figures 2a and b. Different combinations of pump and probe photons therefore produce different binding energy

scales in each case. This can be understood in case of the simultaneous application of the pump and probe beam (Δt=0), which yields an additional contribution to the photoelectron spectrum at lower kinetic energies (see Supplementary Figure 2b, yellow curve, energy range between 0.8 and 2.0 eV) The spectrum can be considered as a superposition of two different ionization processes – one involving two probe photons (equally to solely applying the probe beam; black curve) and, secondly, a mixed transition involving one pump and one probe photon (green curve). The signal intensity dependence of this additional contribution (i.e., the green curve) on the pump pulse intensity is plotted in Supplementary Figure 3 (blue line). The slope of ~ 1.0 provides evidence that the pump beam contributed one photon to this transition. In this example, the use of a single binding energy scale would not render the situation correctly for both spectral contributions.

Both contributions stem from initially occupied states and are, in case of non-existing resonant transitions, mediated via virtual intermediate states. The transient 2PPE signal can be readily corrected for the single-color background (Supplementary Figure 2b, black curve), but the intensity of the two-color photon ionization (Supplementary Figure 2b, green curve) obeys the convolution of the pump and probe temporal envelopes.

In addition, the single-color probe spectrum shown in Supplementary Figure 2b plotted on a linear scale allows determination of the VBM position, by fitting a line to the spectral onset and determining the intersection point of this line with the abscissa. A binding energy 0.5 eV below the Fermi level was found, similar to previously reported values[5,6]. The spectral shape as well as the position of single spectral features was not changed when the probe intensity was varied by more than two orders of magnitude.

## Supplementary Discussion

### Surface band bending

The surface band bending (BB) was evaluated in the $Cu_2O$ semiconductor due to the either adsorption of the terminal molecular oxygen described above as well as due to the formation of a $Cu_2O$/Pt junction. $Cu_2O$ is generally a p-type semiconductor due to the bulk formation of Cu vacancies, with a work function (WF) of 4.9 ± 0.1 eV[7]. For a p-type semiconductor, the bulk Fermi level ($FL_{SC}$) lies closer to the valence band (VB), which is energetically lower than the highest-occupied molecular orbital (HOMO) of the surface adsorbate. Under equilibration of the chemical potentials, the electrons will be transferred from the surface to the bulk, producing a linear electric field across the interface and downward BB. The density of surface states induced by oxygen adsorption (~ $10^{15}$ $cm^{-2}$) is larger than the intrinsic doping level (between $10^8$ and $10^{12}$ $cm^{-2}$ [8]), so the Fermi level of $Cu_2O$ is essentially independent of the bulk doping concentration, and will be pinned by the surface states. An analogous process occurs when Pt is deposited on the semiconductor. The WF of sub-nanometer Pt islands is < 4.9 eV[9], so the $FL_{SC}$ is energetically lower than the FL of the metal ($FL_{Met}$). As a result, at equilibrium of the two FLs, the BB will be increased, in accord with the observed upward binding energy shift of the semiconductor core levels after the deposition of 1 nm of Pt. Supplementary Figures 6a, b display the signals for the Cu 2p and the O 1s core levels, respectively, recorded for the clean, reconstructed $Cu_2O$ (100) surface as well after deposition of 1 nm of Pt.

The constant excitation energy (Al Kα, hv = 1486.7 eV) enables qualitative depth profiling following the methodology first introduced by Kraut et al. through the photoionization of different core levels (i.e. at different photoelectron kinetic energies)[10]. As shown in Supplementary Figure 6a, at a photoelectron kinetic energy of ~ 555 eV (Cu $2p_{3/2}$, binding energy equal to 932.3 eV) the binding energy shift (ΔBE) between the core levels acquired on the bare semiconductor and on the $Cu_2O$/Pt junction was 0.26 eV. In contrast, a ΔBE of 0.44 eV was measured using the O 1s core level spectrum (photoelectron kinetic energy of ~ 955 eV, binding energy = 530.2 eV eV, see Supplementary Figure 6b). Supplementary Figure 7 shows a simulation of the Cu 2p and O 1s core level spectra as a result of the potential profile experienced by the ejected photoelectrons within the semiconductor space-charge (SSC) region convoluted with the corresponding exponential decay of the photoelectron intensity (Supplementary Figure 7a). An exponential decay of the potential within the semiconductor was assumed for the generation of the Cu 2p and O 1s core levels[11]. The simulations were performed considering the BB potential and the SSC width as free parameters, to obtain the same ΔBE for the simulated core levels as that observed experimentally. The inelastic mean free paths (IMFP) were obtained using the Tanuma-Powell-Penn algorithm[12], with a value of 1.2 nm for the Cu $2p_{3/2}$ and 1.8 nm for the O 1s. The corresponding probed volumes (PV) were determined by taking the IMFP times 3, the so-called "3 lambda" value (which "contains" 95% of the overall photoelectron signal). Supplementary Figure 7b shows the normalized simulated core levels, for

both the Cu $2p_{3/2}$ and O 1s regions. The best agreement between the experiments and the simulations was observed for a BB potential of 0.45 V (close to what has been previously found in the literature[7] and a SC width of 7.5 nm, in accord with prior observations[8]. Under these conditions, the simulated ΔBE measured using the Cu $2p_{3/2}$ was 0.32 eV, in good agreement with the experimental value of 0.26 eV. In contrast, the O 1s core level provided a ΔBE value of 0.28 eV, due to the increase of the PV compared to the use of the Cu $2p_{3/2}$ photoionization. In contrast to the situation for the Cu $2p_{3/2}$ core level, the simulated and the experimental ΔBE values are in substantial mutual disagreement. This difference may be due to the simple model used for the simulation of the semiconductor BB, or to a differential charging of the sample. Although based on a simple modeling of the semiconductor BB, the simulations nevertheless allow estimation of the BB potential and SSC width that resulted from deposition of Pt.

Although the XPS data revealed a binding energy shift of the semiconductor core levels (Cu 2p, O 1s), the valence band as well as conduction band position measured by 2PPE were unchanged as a result of Pt deposition. This behavior is consistent with the much higher surface sensitivity of 2PPE experiments relative to XPS. Assuming a band-edge pinning - implying the band edge potentials of the semiconductor are constant at the interface - the 2PPE measurement cannot resolve the changes in band bending, which extend over the complete SC width of 7.5 nm. In contrast, the much higher probing depth of the XPS experiment, and the correspondingly

larger probed volume, provides access to the increase of the BB potential by virtue of the different observed core levels shifts of Cu and O.

For reconstructed samples, XPS did not provide evidence for formation of surface CuO or solid Cu, which are prone to recombination and Schottky barrier formation, respectively,[13–16] both of which may reduce the photovoltage and/or photocurrent.

## Supplementary Methods

### Photoluminescence (PL)

Photoluminescence (PL) measurements were conducted to investigate the origin of the observed defect levels (see Supplementary Figure 12). PL provides bulk information and therefore complements the surface-sensitive 2PPE technique. The experiments were conducted at different stages of sample preparation (reconstructed, Pt-covered) while maintaining UHV conditions throughout. The pump laser pulse conditions were identical to those in the 2PPE measurements. Both PL spectra exhibited a sharp peak at ~ 620 nm that has been assigned to exciton luminescence in $Cu_2O$[17,18] (Supplementary Figure 12a). This peak was used to normalize the spectra, as it is not expected to be influenced by the surface treatment. The second, much broader contribution, in the range between 800 and 1050 nm, can consistently be attributed to defect-mediated radiative recombination. Previously identified defect centers that show PL signals in this range are oxygen vacancies (780 nm) and copper vacancies (930 nm)[19,20]. The PL signal clearly suggests that Cu vacancies are the dominant defect in the samples measured herein. The signal was large compared to the free exciton signal and did not change with different surface treatments, suggesting a relatively high bulk concentration of Cu vacancies. Additionally, for both spectral regions time-resolved PL measurements were conducted and are shown for the

reconstructed surface (Supplementary Figure 12b). After optical excitation the exciton contribution exhibited a fast rise limited by the detector response and exponential fitting revealed a decay constant of $\tau = 1.1$ ns. Previous investigations suggested lifetime limits of around 100 ns[21,22], whereas long lifetimes up to the µs time domain[23] were obtained by using high-quality samples containing only a small amount of copper vacancies. The relatively short lifetime in the present investigation can likewise be seen as a direct consequence of the high copper vacancy concentration as already revealed by the stationary measurements. Complementary to the exciton decay the defect PL rises much slower on a ns time scale and lasts into the µs time domain (not shown here).

**LEED and XPS**

Supplementary Figure 5a shows the low-energy electron diffraction (LEED) data for the reconstructed $Cu_2O$ surface. The LEED measurements show a (1×1) periodicity with ½ monolayer of terminating atomic oxygen adsorbed as a c(2×2) structure (or, equivalently in Wood notation, following a $\sqrt{2} \times \sqrt{2}$ R45° surface pattern), in accord with prior reports[24].

Pt 4f photoemission spectra (XPS) were obtained as a function of increasing Pt thickness, with the Pt deposited via ultra-high vacuum (UHV) evaporation onto the $Cu_2O$ surface (see Supplementary Figure 5b). The bulk Pt–Pt distance along the [111] direction is 0.28 nm and the distance between the top-most layer and the second layer along the [111] direction is 0.23 nm[25]. The resulting thickness-to-monolayer equivalent (MLE) conversion yielded ~ 2 MLE for 0.5 nm Pt coverage, ~ 4 MLE for 1 nm, ~ 54 MLE for 15 nm and ~ 107 MLE for 30 nm.

As shown in Supplementary Figure 5b, the Pt 4f and the Cu 3p core levels overlapped for low coverages (~2 and 4 MLE), whereas contributions from the $Cu_2O$ substrate were completely attenuated for higher Pt coverage. For Pt thicknesses > 1 nm (spectrum C), the Pt 4f photoemission line shape resembled that exhibited by bulk metallic phases (spectra A and B), with a Doniach-Sunjic asymmetry parameter (α) equal to 0.22, as reported previously for analogous systems[26]. In contrast, for the 0.5 nm Pt coverage (spectrum D), the line-shape asymmetry decreased ($\alpha = 0.15$), and was accompanied by a spectral broadening and an upward shift of the 4f binding energy. The changes in the linewidth and line asymmetry are in accord with expectations for final state effects that account for the changes in the response of the valence electrons to the core-hole as a function of the cluster size[26]. The decrease in the line shape asymmetry (i) for low Pt coverage is consistent with expectations for a loss of screening of the core-hole potential[26], whose reduction causes also a decrease of the cluster work function[9] and the consequent positive shift of the 4f binding energy (ii). The linewidth broadening (iii) is instead ascribable to the plasmon excitation at the boundary surface of the cluster[26]. The smaller the Pt cluster, the higher the surface-to-bulk ratio, and the higher the plasmon excitation effect on the acquired spectral signal. The observation of the three aforementioned size effects in the case of the lower Pt coverage is consistent with expectations for a non-conformal Pt overlayer, which presumably consists of separate nanometer-sized islands. A thin, continuous Pt layer exposing under-coordinated Pt atoms may also produce an increase in the 4f binding energy[27]. In contrast,

compared to a bulk Pt phase, the spectral change observed in this latter case typically is characterized by only a shift in binding energy, without modification of the spectral linewidth and asymmetry. The fitting procedure also did not yield evidence for oxidized Pt species at the semiconductor/metal interface.

To summarize, X-ray photoelectron spectroscopy (XPS) data and its theoretical modeling indicated a non-conformal growth for Pt deposition via UHV evaporation of films with thicknesses < 1 nm. For films with theoretical thickness equivalent to 1 nm and larger – as determined by monitoring the rate of deposition using a quartz crystal microbalance (QCM) in UHV – the fitting procedure of the XPS data revealed a decrease in the size effects (line shape asymmetry, shift of the Pt 4f binding energy, linewidth broadening) associated with non-conformal growth (Supplementary Figure 5b). Based on these results, Pt films with QCM thickness equivalent to 1 nm were used for all of the experiments discussed herein.

**AFM+SEM/EDX**

Atomic force microscope (AFM, NT-MDT Ntegra) images showed a non-conformal structure after Pt deposition on the reconstructed $Cu_2O$ (100) surface (see Supplementary Figure 8). Non-laminar island growth is often observed for vacuum metal deposition on metal oxides because of weak adatom-substrate interactions[28]. Three-dimensional clusters with diameters of ~ 1 μm and heights of ~ 3 nm were distributed on the $Cu_2O$ (100) surface. In addition, island

growth was centered around pinholes that extended tens of nm into the $Cu_2O$ (100) surface. This type of growth behavior has been previously observed and ascribed to a trapping of adatoms at surface defects, thereby forming nuclei for a subsequent heterogeneous nucleation growth process[29]. The AFM images were obtained in semicontact mode and the phase signal showed a clear contrast that resembled the domain boundaries observed in the height map. This correlation is consistent with the presence of different mechanical properties, and hence different materials, inside and outside the domains.

The presence of Pt islands on the $Cu_2O$ surface was verified by means of scanning-electron microscope (SEM) imaging and EDX elemental distribution mapping (Supplementary Figure 9), performed at a beam energy of 2 kV to minimize the excitation volume. Data were obtained using a Zeiss UltraPlus scanning electron microscope and an Oxford Instruments Ultim Extreme EDX system with Aztec software suite, at a beam current of ~ 10 nA for optimal signal-to-noise ratio). The Pt-N and C-K lines are very close in energy to each other, thus, the EDX data alone cannot unambiguously confirm the presence of Pt in the islands. However, additional SEM imaging at much lower beam current (about 10 pA) than for the SEM image in Supplementary Figure 9, indicated that the islands were composed of small agglomerates (about 100 nm in diameter), which is typical for Pt but not for C (Supplementary Figure 10).

**Supplementary References**


1. Hertel, T., Knoesel, E., Wolf, M. & Ertl, G. Ultrafast electron dynamics at Cu (111): Response of an electron gas to optical excitation. *Phys. Rev. Lett.* **76**, 535–538 (1996).

2. Knoesel, E., Hotzel, A. & Wolf, M. Ultrafast dynamics of hot electrons and holes in copper: Excitation, energy relaxation, and transport effects. *Phys. Rev. B* **57**, 12812–12824 (1998).

3. Lisowski, M. *et al.* Ultra-fast dynamics of electron thermalization, cooling and transport effects in Ru (001). *Appl. Phys. A* **78**, 165–176 (2004).

4. Elliott, R. J. Symmetry of excitons in $Cu_2O$. *Phys. Rev.* **124**, 340–345 (1961).

5. Deuermeier, J., Gassmann, J., Brötz, J. & Klein, A. Reactive magnetron sputtering of $Cu_2O$: Dependence on oxygen pressure and interface formation with indium tin oxide. *J. Appl. Phys.* **109**, 113704 (2011).

6. Ichimura, M. & Song, Y. Band alignment at the $Cu_2O$/ZnO heterojunction. *Jpn. J. Appl. Phys.* **50**, 051002 (2011).

7. Kwon, J.-D. *et al.* Controlled growth and properties of p-type cuprous oxide films by plasma-enhanced atomic layer deposition at low temperature. *Appl. Surf. Sci.* **285**, 373–379 (2013).

8. Zhang, Z. & Yates, J. T. Band bending in semiconductors: Chemical and physical consequences at surfaces and interfaces. *Chem. Rev.* **112**, 5520–5551 (2012).

9. Zheng, H., Zhou, Y. & Gangopadhyay, S. Size-dependent work function and single electron memory behavior of pentacene non-volatile memory with embedded sub-nanometer

platinum nanoparticles. *J.Appl. Phys.* **117**, 024504 (2015).

10. Kraut, E. A., Grant, R. W., Waldrop, J. R. & Kowalczyk, S. P. Precise determination of the valence-band edge in X-ray photoemission spectra: Application to measurement of semiconductor interface potentials. *Phys. Rev. Lett.* **44**, 1620–1623 (1980).

11. Memming, R. *Semiconductor Electrochemistry*. (Wiley-VCH, Weinheim, 2015).

12. Tanuma, S., Powell, C. J. & Penn, D. R. Calculations of electron inelastic mean free paths. *Surf. Interface Anal.* **37**, 1–14

13. Sears, W. M. & Fortin, E. Preparation and properties of $Cu_2O$/Cu photovoltaic cells. *Sol. Energy Mater.* **10**, 93–103 (1984).

14. Olsen, L. C., Bohara, R. C. & Urie, M. W. Explanation for low-efficiency $Cu_2O$ Schottky-barrier solar cells. *Appl. Phys. Lett.* **34**, 47–49 (1979).

15. Lee, S. W. *et al.* Improved $Cu_2O$-based solar cells using atomic layer deposition to control the Cu oxidation State at the p-n junction. *Adv. Energy Mater.* **4**, 1301916 (2014).

16. Siol, S. *et al.* Band alignment engineering at $Cu_2O$/ZnO heterointerfaces. *ACS Appl. Mater. Interfaces* **8**, 21824–21831 (2016).

17. Ito, T. & Masumi, T. Detailed examination of relaxation processes of excitons in photoluminescence spectra of $Cu_2O$. *J. Phys. Soc. Jpn.* **66**, 2185–2193 (1997).

18. Li, J. *et al.* Engineering of optically defect free $Cu_2O$ enabling exciton luminescence at room temperature. *Opt. Mater. Express, OME* **3**, 2072–2077 (2013).

19. Solache-Carranco, H. *et al.* Photoluminescence and X-ray diffraction studies on $Cu_2O$. *J. Lumin.* **129**, 1483–1487 (2009).

20. Harukawa, N. *et al.* Temperature dependence of luminescence lifetime in $Cu_2O$. *J. Lumin.* **87**, 1231–1233 (2000).

21. Snoke, D. W., Shields, A. J. & Cardona, M. Phonon-absorption recombination luminescence of room-temperature excitons in $Cu_2O$. *Phys. Rev. B* **45**, 11693–11697 (1992).

22. Koirala, S., Naka, N. & Tanaka, K. Correlated lifetimes of free paraexcitons and excitons trapped at oxygen vacancies in cuprous oxide. *J. Lumin.* **134**, 524–527 (2013).

23. Mysyrowicz, A., Hulin, D. & Antonetti, A. Long exciton lifetime in $Cu_2O$. *Phys. Rev. Lett.* **43**, 1123–1126 (1979).

24. Schulz, K. H. & Cox, D. F. Photoemission and low-energy-electron-diffraction study of clean and oxygen-dosed $Cu_2O$ (111) and (100) surfaces. *Phys. Rev. B* **43**, 1610–1621 (1991).

25. Kitchin, J. R., Nørskov, J. K., Barteau, M. A. & Chen, J. G. Modification of the surface electronic and chemical properties of Pt (111) by subsurface 3d transition metals. *J. Chem. Phys.* **120**, 10240–10246 (2004).

26. Cheung, T. T. P. X-ray photoemission of small platinum and palladium clusters. *Surf. Sci.* **140**, 151–164 (1984).

27. Ogasawara, H. *et al.* Structure and bonding of water on Pt (111). *Phys. Rev. Lett.* **89**, 276102 (2002).

28. Chambers, S. A., Droubay, T., Jennison, D. R. & Mattsson, T. R. Laminar growth of ultrathin metal films on metal oxides: Co on hydroxylated $\alpha$-$Al_2O_3$ (0001). *Science* **297**, 827–831 (2002).

29. Bäumer, M. & Freund, H.-J. Metal deposits on well-ordered oxide films. *Prog.Surf. Sci.* **61**, 127–198 (1999).
